# Evidence‐based Clinical Practice Guideline on the Use of Blood‐based Biomarkers in Alzheimer’s Disease: Presentation of Preliminary Evidence, and Opportunity for Public Comment

**DOI:** 10.1002/alz.095319

**Published:** 2025-01-09

**Authors:** Oskar Hansson, Heather Whitson, Rebecca M. Edelmayer, Laura A Allen, Douglas R. Galasko, Thomas K Karikari, Hamid Okhravi, Madeline Paczynski, Suzanne E. Schindler, Marc Suarez‐Calvet, Charlotte Teunissen, Henrik Zetterberg, Simin Mahinrad, Sarah Pahlke, Mary Beth McAteer, Lara Kahaleh, Malavika Tampi

**Affiliations:** ^1^ Lund University, Lund Sweden; ^2^ Duke University, Durham, NC USA; ^3^ Alzheimer’s Association, Chicago, IL USA; ^4^ Mayo Clinic, Rochester, MN USA; ^5^ University of California, San Diego, La Jolla, CA USA; ^6^ University of Pittsburgh, Pittsburgh, PA USA; ^7^ Eastern Virginia Medical School, Norfolk, VA USA; ^8^ Washington University School of Medicine, Saint Louis, MO USA; ^9^ Washington University in St. Louis School of Medicine, St. Louis, MO USA; ^10^ Barcelonaβeta Brain Research Center (BBRC), Pasqual Maragall Foundation, Barcelona Spain; ^11^ Amsterdam University Medical Centre, Amsterdam Netherlands; ^12^ University of Gothenburg, Gothenburg Sweden; ^13^ Infectious Diseases Society of America, Arlington, VA USA; ^14^ Virginia Mason Franciscan Health, Seattle, WA USA

## Abstract

**Background:**

The rapid development of blood‐based biomarkers (BBMs) has improved Alzheimer’s disease (AD) diagnostics with some tests now potentially suitable for clinical use. This aligns with the clinical availability of anti‐Aβ immunotherapies for early symptomatic AD, where BBMs can help address the increasing need for more rapid and early diagnosis. Clinical practice guidelines (CPG) can help guide healthcare professionals in incorporating BBMs into their clinical practice. The Alzheimer’s Association is collaborating with methodologists and a panel of 11 clinical and subject‐matter experts to develop a CPG for specialists, informed by a systematic review, on the use of BBMs for AD.

**Method:**

The panel drafted several clinical questions prioritizing two questions on the use of BBMs in cognitively impaired or cognitively unimpaired individuals as the populations of immediate interest. Plasma p‐tau and Aβ biomarkers tests are *index tests*, and amyloid PET imaging, neuropathology, and CSF are *reference standards*. Experts in the GRADE (Grading of Recommendations, Assessment, Development, and Evaluations) approach are conducting a systematic review of the diagnostic accuracy of BBMs index tests by searching three databases (Pubmed, Embase, Cochrane Library) and will include assessments of the quality of collected evidence to inform the panel’s decision‐making process. Patients/caregivers and key external organizations will be engaged throughout.

**Result:**

This session will cover *preliminary* evidence for answering the two clinical questions. At the time of abstract submission, the search strategy resulted in 880 titles/abstracts for the study selection process. Data abstraction, analysis, and evidence development are pending. Recommendations for answering clinical questions will be developed after July 2024, and all recommendations will be finalized and published by early 2025.

**Conclusion:**

The integration of BBMs into clinical care offers the potential to improve earlier and more accurate AD diagnosis for a broader patient population and expand access to new AD therapies. The development of evidence‐based CPGs is a critical step forward in optimizing and standardizing the clinical use of BBMs. As the field of AD diagnostics and treatment continues to evolve, this CPG on the use of BBMs for AD will expand and be regularly updated to include new target audiences/settings, patient populations, and associated evidence.

